# Characterization of a Chinese Hamster Ovary Cell Mutant Having a Mutation in Elongation Factor-2

**DOI:** 10.1371/journal.pone.0009078

**Published:** 2010-02-05

**Authors:** Pradeep K. Gupta, Shihui Liu, Stephen H. Leppla

**Affiliations:** Laboratory of Bacterial Diseases, National Institute of Allergy and Infectious Diseases, National Institutes of Health, Bethesda, Maryland, United States of America; University of Hong Kong, Hong Kong

## Abstract

Retroviral insertional mutagenesis provides an effective forward genetic method for identifying genes involved in essential cellular pathways. A Chinese hamster ovary cell line mutant resistant to several bacterial ADP-ribosylating was obtained by this approach. The toxins used catalyze ADP-ribosylation of eukaryotic elongation factor 2 (eEF-2), block protein synthesis, and cause cell death. Strikingly, in the CHO PR328 mutant cells, the eEF-2 substrate of these ADP-ribosylating toxins was found to be modified, but the cells remained viable. A systematic study of these cells revealed the presence of a structural mutation in one allele of the eEF-2 gene. This mutation, Gly717Arg, is close to His715, the residue that is modified to become diphthamide. This Arg substitution prevents diphthamide biosynthesis at His715, rendering the mutated eEF-2 non-responsive to ADP-ribosylating toxins, while having no apparent effect on protein synthesis. Thus, CHO PR328 cells are heterozygous, having wild type and mutant eEF-2 alleles, with the latter allowing the cells to survive even in the presence of ADP-ribosylating toxins. Here, we report the comprehensive characterization of these cells.

## Introduction

Diphthamide is a post-translationally modified histidine residue found exclusively in eukaryotic translation elongation factor 2 (eEF-2) [1]–[3]. Cellular eEF-2 plays an essential role in translation by mediating the movement of the transfer-RNA bound nascent polypeptide chain between sites on the ribosome after addition of each amino acid [4]. The diphthamide side chain is the target of several potent bacterial toxins, specifically diphtheria toxin (DT), *Pseudomonas* exotoxin A (ETA), cholix toxin of *Vibrio cholerae*, and FP59, an anthrax toxin-derived fusion protein extensively used in this laboratory [5]–[9]. These toxins exert their toxic effect by catalyzing transfer of the ADP-ribose portion of NAD to diphthamide, a modification that inactivates eEF-2 and leads to cell death [5]. The biosynthesis of diphthamide is complex, requiring at least five proteins to catalyze three steps in construction of this post-translational modification of His715 in eEF-2 [10]. This modification and the five biosynthetic proteins are conserved in all eukaryotes, pointing to an important but still elusive role for diphthamide in normal physiology.

DT and ETA were the first two ADP-ribosylating toxins identified and biochemically characterized [11], [12]. DT is produced as a single polypeptide that requires cleavage into two fragments. Fragment A contains the catalytic domain whereas fragment B contains the receptor-binding domain and a region involved in membrane translocation. DT binds to a specific receptor, the heparin-binding epidermal growth factor-like precursor, enters into the cytosol by receptor-mediated endocytosis, and translocates from acidified endosomes to the cytosol, where it inactivates eEF-2 [7], [11]. On the other hand, ETA is a single polypeptide chain which has an amino-terminal receptor-binding domain, a central membrane translocation domain and a carboxyl-terminal catalytic domain [12], [13]. ETA uses the low-density lipoprotein receptor-related protein (LRP1) to enter cells by endocytosis, and then trafficks by a retrograde route to the endoplasmic reticulum to eventually reach the cytosol [13]. The newly discovered cholix toxin of *Vibrio cholerae* also has the same enzymatic activity, but little is known about its receptor and the mechanism by which it reaches the cytosol [8].

This laboratory has exploited the potency of the ADP-ribosylating toxins as a tool in characterizing the anthrax toxins. Thus, we routinely use a chimeric toxin termed fusion protein 59 (FP59), which utilizes the anthrax toxin receptor and translocation mechanism to deliver the ADP-ribosylating domain of ETA to the cytosol of cells [6]. Anthrax toxin is comprised of three proteins, protective antigen (PA), lethal factor (LF) and edema factor (EF) [14]. The combination of PA and LF is termed lethal toxin (LT). PA binds to either the tumor endothelium marker 8 (TEM8) or capillary morphogenesis gene product 2 (CMG2) proteins on the cell surface and causes the endocytosis and trafficking of LF and EF to the cytosol of host cells [14]. The toxicity of LT is due to the catalytic activity of LF, a protease which cleaves several MAP kinase kinases [15]. In FP59, the amino-terminal 254 residues of lethal factor, which constitutes the domain responsible for binding to PA, are fused with the catalytic domain of ETA [6], [16]. FP59 administered with PA is toxic to mammalian cells due to delivery of FP59 to the cytosol. The receptors for PA are widely distributed, making the PA + FP59 combination lethal to almost all cell types tested. Based on classical studies with DT [17], it is expected that a single molecule of these toxins can inactivate a cell's entire supply of eEF-2 within hours, bringing protein synthesis to a halt and leading to cell death.

In earlier studies that sought to identify the anthrax toxin receptor, retroviral insertional mutagenesis was performed with CHO WTP4 (i.e., parental) cells, and clones were selected for resistance to PA + FP59 [18]. Mutants were initially classified into two groups - one group having defects in toxin uptake and another group that were cross-resistant to other ADP-ribosylating toxins [18]. The uptake mutants included CHO PR230, which was shown to lack a functional toxin receptor [19]. Most of the latter, cross-resistant mutants were found to have defects in the synthesis of diphthamide, the unique modified His residue in eEF-2 that is the target of ADP-ribosylation. One mutant cell line from the second group, CHO PR328, showed less sensitivity to all the ADP-ribosylating toxins, DT, ETA, and FP59. However, the CHO PR328 mutant cells were found to differ from other mutants in that the eEF-2 of these cells were sensitive to toxin-induced ADP-ribosylation. We performed a systematic study to characterize these cells, as reported in the present work.

## Results

### Toxin Induced Cytotoxicity and ADP-Ribosylation of eEF-2 in CHO PR328 Cells

**Figure 1:**
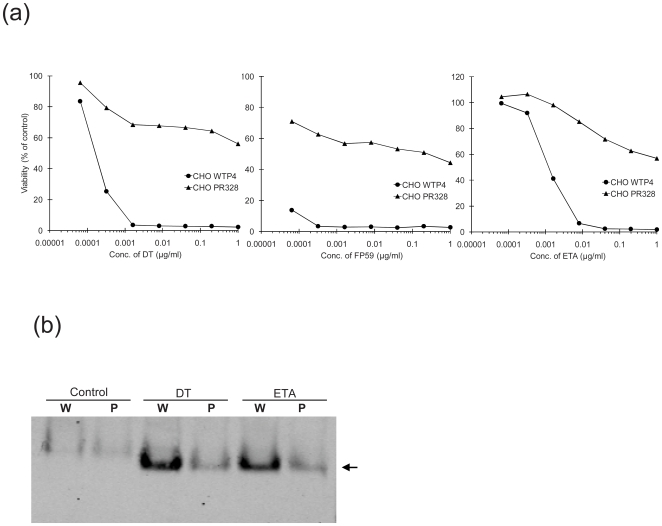
Toxin induced cytotoxicity and ADP-ribosylation of eEF-2. (**A**) CHO PR328 and their parental cells, CHO WTP4, were incubated with the indicated concentrations of toxins (in case of toxicity to FP59, PA was also added at a fixed concentration of 500 ng/ml). Cell viability was assessed at 48 h by the MTT assay. The A_540_ values obtained for cells that received no toxin were considered as 100 % and used to calculate the percent viability of other data points. (**B**) Cells were treated with 100 ng/ml of diphtheria toxin (DT) or Pseudomonas exotoxin A (ETA) for 4 h and the cell lysate was tested for the presence of ADP-ribosylated eEF-2. Equal amount of cell lysate was subjected to native PAGE followed by western blotting with anti-eEF-2 antibodies. As discussed in ‘Results’, these antibodies react more strongly with the ADP-ribosylated form than with the unmodified EF-2. W and P symbolize CHO WTP4 and CHO PR328 cells respectively. Arrow indicates the ADP-ribosylated eEF-2.

CHO PR328 cells were obtained from parental CHO WTP4 cells following selection with a combination of anthrax PA and fusion protein FP59 [18]. In cytotoxicity assays, >50% of CHO PR328 cells survived even very high concentrations of the ADP-ribosylating toxins DT, ETA and fusion toxin PA + FP59, while parental CHO WTP4 cells were highly sensitive to these toxins (Fig. 1a).

To determine whether the CHO PR328 cells contain eEF-2 that can be ADP-ribosylated, the cells were treated with DT or ETA for 1 h and then cell lysates were separated on native PAGE followed by western blotting with anti-eEF-2 antibodies. We previously showed that the added negative charge of ADP-ribose causes ADP-ribosylated eEF-2 to migrate faster than unmodified eEF-2 on native PAGE [18]. The eEF-2 in toxin-treated CHO PR328 cells was ADP-ribosylated in a manner similar to that in the parental cells (Fig. 1b). This result was surprising, since the modified eEF-2 cannot function in protein synthesis, yet these cells survived. This analysis also seemed to show that there was less ADP-ribosylated eEF-2 in the CHO PR328 lysates than in the parental cell lysates. However, as we noted previously [10], the antibody used here reacts more strongly to the modified eEF-2 than to the native protein (compare the “W” bands in control vs. toxin treated lanes), so that the relative amounts of the two forms cannot be estimated from images like that in Fig. 1b.

### Validation of Toxin Internalization in CHO PR328 Cells

**Figure 2:**
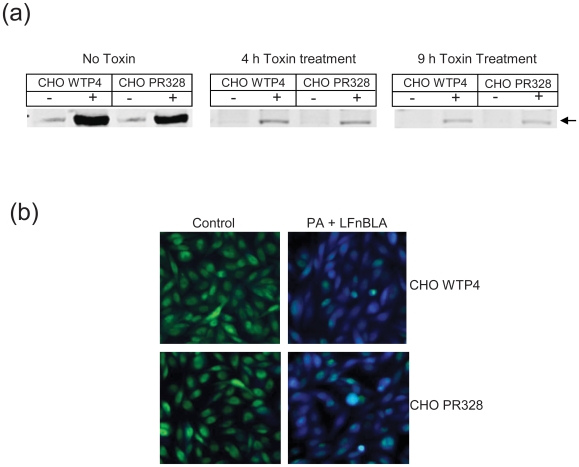
Toxin internalization in CHO PR328 cells. (**A**) Cells were treated with PA and FP59 (1 µg/ml each) for 1 h. Then toxin was replaced with fresh media after three washes with fresh medium, and incubated an additional 3 h or 8 h. At the end of desired incubation, cell lysates were prepared and tested for DT-induced ADP-ribosylation *in vitro* using biotin-NAD. Equal amounts of protein were then separated by SDS-PAGE followed by western blotting with streptavidin conjugated to Alexa Fluor dye and membrane was scanned on an Odyssey Infrared Imager. Left panel shows the control cells that did not receive treatment with PA + FP59. Middle panel shows the data with cells treated with PA + FP59 for 4 h (1 h treatment + additional 3 h) while right panel is for cells treated with PA + FP59 for 9 h (1 h treatment + additional 8 h). – and + denote the absence or presence of DT during the in vitro ADP-ribosylation reaction. Arrow indicates the biotin-ADP-ribose-eEF-2. (**B**) Cells were treated with PA and LFnBLA (1 µg/ml each) for 1 h. Thereafter, 1.5 µM CCF2/AM was added to the cells for 1 h at room temperature. The CCF2/AM remaining in the medium was removed by washing and cells were incubated an additional 1 h at room temperature to allow for CCF2/AM hydrolysis. The cells were then photographed with a fluorescence microscope using an excitation wavelength of 405 nm and emission filters of 530 nm (green light) and 460 nm (blue light). Beta-lactamase activity in treated cells is demonstrated by the blue fluorescence while control cells show green fluorescence.

While the data in Fig. 1b suggested that the DT and ETA toxins trafficked normally to the cytosol in CHO PR328, it was also important to consider the alternative hypothesis that the ADP-ribosylation seen in Fig. 1b was due to artifactual modification of eEF-2 occurring after lysis of the cells. The RIPA buffer used for the cell lysis is known to rupture all intracellular vesicles and it does not contain inhibitors that would prevent ADP-ribosylation catalyzed by toxin that was trapped in and only released from vesicular compartments upon cell lysis. To examine this hypothesis, cells were exposed to PA + FP59 for 1 h and then incubated without toxin for additional periods of either 3 or 8 h. After these times, cell lysates were prepared and the presence of any remaining non-ribosylated eEF-2 was detected by addition of biotin-NAD along with additional DT. If the FP59 trafficked normally to the cytosol and ADP-ribosylated the eEF-2, then unmodified eEF-2 would no longer be available for *in vitro* ADP-ribosylation. If in contrast, the FP59 was trapped in vesicular compartments and degraded over time, then it would produce more biotin-ADP-ribose-eEF-2 at early than late times. The former result was observed (Fig. 2a), since only a trace of ADP-ribosylation was found, and this decreased with time of toxin treatment of the cells. This shows that the modification of eEF-2 occurred prior to preparation of the lysates and was not artifactual. We also estimated the amount of toxin-catalyzed ADP-ribosylated eEF-2 in the “no toxin” controls (from Fig. 2a left panel) using Odyssey software. The background corrected intensities of ADP-ribosylated eEF-2 were 7519 and 4102 units for CHO WTP4 and CHO PR328 cells, respectively, indicating ∼50% less ADP-ribosylated eEF-2 in CHO PR328 cells than in parental cells. Intensities of bands obtained by western blotting with eEF-2 antibodies were nearly the same for both cells (data not shown).

To directly examine the internalization process, LFnBLA was used to visualize the uptake in CHO PR328 cells. LFnBLA is a fusion protein of LF amino acids 1-254 and β-lactamase. When used in combination with PA, this fusion protein is translocated to the cytosol of mammalian cells, where it's catalytic activity can be visualized using a fluorescent substrate, CCF2/AM [20], [21]. This membrane permeable substrate becomes both trapped in cells and susceptible to cleavage by β-lactamase due to removal of the acetoxy groups by cytosolic esterases. An intact molecule of CCF2/AM emits at 520 nm (green light) when excited at 409 nm, as a result of an intramolecular fluorescence resonance energy transfer (FRET) between 7-hydroxycoumarin and fluorescein. Hydrolysis of CCF2/AM by β-lactamase liberates the acceptor fluorescein and leads to fluorescence at 447 nm (blue light) by the donor coumarin. Thus, cells in which the LFnBLA fusion protein reaches the cytosol show a shift of the initial green fluorescence to blue. To determine whether LFnBLA (and by inference LF and FP59) is internalized in CHO PR328, cells were incubated with 2 µg/ml each of PA and LFnBLA for 2 h, washed, loaded with substrate for 2 h, and then imaged (Fig. 2b). Successful delivery of LFnBLA into CHO PR328 and CHO WTP4 cells was evident by the bright blue fluorescence of treated but not control cells. Several other studies using PA mutants and inhibitors to provide additional evidence about the normal uptake of toxin in CHO PR328 cells have been included in supplementary materials (File S1, Fig. S1 and Fig. S2).

### Genotyping of eEF-2 Alleles

As described above, biochemical characterization of CHO PR328 cells ruled out the presence of defects in toxin binding, internalization, and trafficking. Instead, the data demonstrated that toxin was internalized in CHO PR328 cells and successfully ADP-ribosylated the eEF-2. In reviewing the available literature, we recognized that previous reports had identified mutated eEF-2 variants which lack diphthamide but retain some degree of normal function in translation. One such mutation, Gly717Arg, has been observed in several previous studies [22]–[24] and has therefore been considered an apparent mutational “hot spot”. The nearby His715 in this mutated eEF-2 cannot be modified to diphthamide, apparently due to a structural perturbation that prevents recognition by the diphthamide biosynthesis enzymes. Several other reported, but less frequent, mutations in this region that block diphthamide synthesis include Gly719Arg, Ile714Asn, and Ser584Gly. Most of these residues are located in the strictly conserved region around the diphthamide site in eEF-2 (Fig. S3). It seemed reasonable to consider that CHO PR328 cells might be heterozygous for one of these mutations, which could lead to the observed dominant toxin-resistance phenotype.

**Figure 3:**
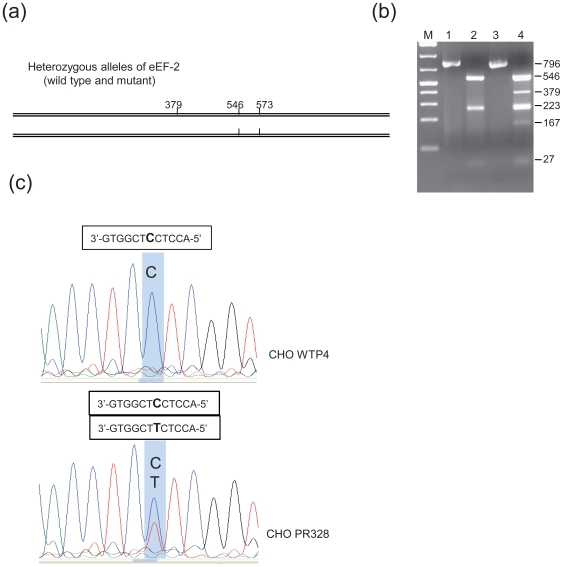
Genotyping of eEF-2 from CHO WTP4 and CHO PR328 cells. (**A**) Schematic representation for the presence of MboII restriction sites in heterozygous alleles in a 796-bp eEF-2 PCR product. Double lines indicate the two alleles of eEF-2 while vertical small lines show the sites for MboII restriction enzymes. There are two sites in wild type eEF-2 (bottom line) while the G to A transition introduces one more site in the mutant allele (top line). (**B**) RNA was extracted from CHO WTP4 and CHO PR328 cells using TRIzol reagent. RT-PCR was performed and amplified product was digested with MboII restriction enzyme. Digested DNA samples along with controls were then separated on 1% agarose gel and visualized using UV gel documentation system. M: 1 kb DNA ladder; Lane 1: CHO WTP4 control; Lane 2: CHO WTP4 digested; Lane 3: CHO PR328 control; and Lane 4: CHO PR328 digested. Presence of 5 bands is evident for CHO PR328 cells while CHO WTP4 cells shows only 3 bands on DNA gel. (**C**) RT-PCR samples obtained for the analysis of panel B were sequenced. Sequence shown is of complementary strand as reverse primer was used to sequence the region. Zoomed image shows the presence of two peaks (C & T) in eEF-2 of CHO PR328 cells as opposed to only one peak (C) in CHO WTP4 cells in the same region revealing the presence of G & A bases in eEF-2 of CHO PR328 at this location. As expected, CHO WTP4 eEF-2 had only G base at same location.

A Gly717Arg mutation can result from a single bp change of G to A at the first base of codon717 in the eEF-2 gene. Conveniently, this change creates a diagnostic MboII restriction site [24]. To determine whether CHO PR328 cells have this mutation, we performed RT-PCR and restriction digestion with MboII. Fig. 3a shows the schematic representation of the possible eEF-2 alleles. As shown, there are two MboII restriction sites at bp 546 and 573 in the 796-bp amplified fragment from wild type eEF-2 cDNA. If present, the mutation introduces an additional site at position 379 in the mutant eEF-2 gene. Upon restriction digestion with MboII, homozygous wild type alleles will yield 3 fragments of 27, 223 and 546 bp, while homozygous mutant alleles will give 4 fragments of 27, 167, 223 & 379 bp, and, finally, heterozygous alleles should give all 5 fragments of 27, 167, 223, 379 & 546 bp. Genotyping of the eEF-2 gene in this way revealed that CHO PR328 cells are indeed heterozygous for this eEF-2 mutation (Fig. 3b). The heterozygous nature of the eEF-2 alleles and the presence of the G to A transition in CHO PR328 cells were further confirmed by sequencing of the RT-PCR product(s) (Fig. 3c). Thus, the ADP-ribosylation observed in the cells occurs on the wild type eEF-2 protein, thereby inactivating it, while the mutant eEF-2 remains active and is sufficient to support protein synthesis. This finding is consistent with previous reports that cells can survive if they have translation rates as low as 20–30% of normal [22], [24].

### Effect of Toxin Exposure on Subsequent Responses to Toxin

**Figure 4:**
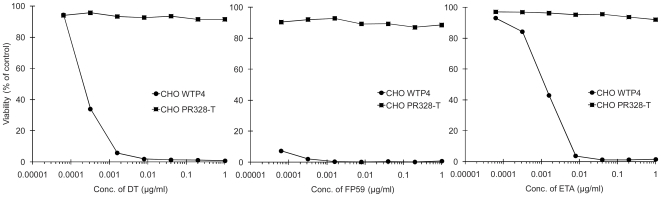
Effect of toxin exposure on CHO PR328 cells for subsequent cytotoxicity to toxin. CHO PR328 cells were treated with PA + FP59 (1 µg/ml) for 48 h and then placed into fresh medium lacking toxin. Cells grown for an additional two days are designated CHO PR328-T. In toxicity assays, cells were treated with different concentrations of FP59 at a fixed concentration of PA (500 ng/ml) for 48 h and then MTT assay was performed to measure cell viability. CHO WTP4 cells were included as control in the experiment.

We further characterized CHO PR328 cells to analyze their growth characteristics. In the presence of toxin, CHO PR328 cells exhibited reduced growth as confirmed by phase-contrast microscopy (data not shown). To examine the long-term response of the cells, they were treated with PA plus FP59 for 48 h (as in Fig. 1) and then grown further in fresh toxin-free medium. After an additional two days of growth, these cells were assayed for their sensitivity to eEF-2 ADP-ribosylating toxins (Fig. 4). The cells showed 100% resistance to toxin, and thus behaved as if they did not receive a second toxin treatment (Fig. 4). This argues that all the wild type eEF-2 in these cells remain fully ADP-ribosylated due either to the earlier treatment with toxin or to the survival of catalytically active toxin in the cytosol. Thus, the newly added toxin could have no further impact on the growth or survival of the cells. When this experiment was extended to longer time intervals, the resistance of the toxin-treated cells returned to the original 50% level after one week of growth (data not shown, similar to Fig. 1), indicating that the toxin had decayed and that the ADP-ribosylated wild type eEF-2 is eventually replaced by newly synthesized wild type eEF-2.

## Discussion

We previously used retroviral insertional mutagenesis to identify genes involved in the action of anthrax toxin on cells [18], [19]. In one approach, CHO cells transfected with the receptor for avian leukosis virus (ALV) were treated with an ALV vector and then selected with PA plus the fusion protein FP59. One group of the resulting toxin-resistant mutants lacked functional PA receptors and showed resistance to PA plus FP59, but retained sensitivity to DT and ETA (e.g., CHO PR230; [19]). Another group of mutants showed resistance not only to PA plus FP59 but also to DT and ETA, suggesting that the cells were altered in genes affecting eEF-2, the target shared by these three toxins. One mutant cell line from the latter group was found to have a disruption of a small gene, DESR1 (later renamed Dph3), that is required for the biosynthesis of diphthamide [10], [18]. The CHO PR328 cells discussed in this report were in the second group of mutant cells which showed less sensitivity to the ADP-ribosylating toxins. These mutant cells are different from earlier reported mutant cells as the eEF-2 of these cells are sensitive to toxin-induced ADP-ribosylation while the cells show reduced sensitivity. We performed several studies (FP59-induced ADP-ribosylation and effect of well established mutants and inhibitors; imaging of cells after delivery of fusion protein LFnBLA, and activity of anthrax lethal toxin) to characterize the internalization of toxin in these cells and all the results indicated that the cells had normal toxin binding and internalization (see supporting information, File S1).

Southern blotting showed that CHO PR328 cells did not contain any retroviral insertions (data not shown) indicating the presence of a spontaneous mutation in these cells. Given the large size of the mammalian genome, any random forward mutational selection must examine a very large number of cells to find the rare mutation that causes the desired phenotype. Applying a phenotypic selection to such a large population will inevitably lead to simultaneous selection of spontaneous mutations, which is clearly what occurred in the present case.

Several previous reports described structural gene mutations in eEF-2 which block the post-translational modification of His715 to diphthamide without seriously decreasing the ability of eEF-2 to participate in protein synthesis [22]–[24]. One of these mutations, Gly717Arg, was described in prior reports [22]–[24]. Restriction analysis confirmed the presence of an Arg717 codon in one of the alleles of eEF-2 in CHO PR328 cells. Sequencing the gene further confirmed that CHO PR328 cells are heterozygous, with one allele being wild type and the other having the Arg717 codon. This residue is in close proximity to the His715 that is normally modified to diphthamide. Substitution of this residue with Arg abolishes the modification of His715 to diphthamide, making this mutant eEF-2 resistant to the toxins. Surprisingly, this mutation has no detectable impact on protein synthesis in cells. Thus, the mutant allele allows the survival of CHO PR328 cells in the presence of toxin, as only 50% of the cellular eEF-2 is inactivated by the toxin while the other 50% eEF-2 remains functional due to, and in spite of, the absence of diphthamide. The survival and continued proliferation of these cells suggests that the ADP-ribosylated and inactive eEF-2 does not strongly compete with mutant (Arg717) eEF-2 for the protein synthesis machinery. However, some inhibitory effect cannot be ruled out, because cells can survive even at 20–30% of the normal translation rate [24].

The amino acid sequence of eEF-2 around His715 and the presence of the diphthamide side chain are both conserved in all eukaryotes (Fig. S3). This probably reflects the multiple functional roles played by this region – recognition by the enzymes that construct the diphthamide side chain, the apparent gain in normal function conferred by diphthamide [25], and participation by the proximal regions (even in the absence of diphthamide) in the elongation step on ribosomes. These multiple structural requirements limit the opportunities for creation of mutations that lack diphthamide while retaining function in protein synthesis. Thus, it is not surprising that only a few such mutations have been reported. The mutations other than those presented here which can provide such resistance are Gly719Arg, Ile714Asn, and Ser584Gly, but these appear to be less frequent. Most of these mutations (except Ser584) lie in the highly conserved diphthamide loop. In fact, Ser584 is located close to His715 in the three-dimensional structure of EF-2 of other species [24], suggesting an important role of this residue. The lower frequency of selection of other mutants may reflect a decreased ability of other eEF-2 variants to support protein synthesis, thereby causing a greater growth defect, and decreasing the chance that a colony will grow big enough to be detected and picked. We speculate that the Gly717Arg mutation is favored because it provides a positive charge that mimics that of diphthamide. In this view, substitution of Gly717 to Arg prevents the diphthamide biosynthesis but at the same time provides a “replacement” positive charge in this region which is sufficient to confer some degree of functionality. This hypothesis about the role of positive charge in the region needs further anylsis. If diphthamide-deficient mutant eEF-2 proteins function perfectly well, it then becomes difficult to understand the retention of the diphthamide modification in such a diverse set of species, which argues strongly for its essentiality.

## Materials and Methods

### Materials

PA, LF, FP59, DT, ETA, and PA mutant proteins were produced in our laboratory as described previously [26]–[28]. LFnBLA, consisting of the N-terminal region of LF (LFn) and β-lactamase (BLA), was produced as previously described [20]. Anti-eEF-2 antibodies specific to the carboxy-terminus of eEF-2 of human origin were purchased from Upstate (Now Millipore, Billerica, MA) and Santa Cruz Biotechnology (Santa Cruz, CA), respectively.

### Cell Culture and Cytotoxicity Assays

Cells were grown in α-minimal essential medium (MEM) supplemented with 8% fetal bovine serum, 25 mM HEPES, 2 mM glutamine and 50 µg/ml gentamicin (all obtained from Invitrogen) at 37°C in 5% CO2. For cytotoxicity assays, cells were sub-cultured in 96-well plates one day prior to experiments as described previously [9]. Cells were treated with various concentrations of toxins for 48 h and cell viability was measured by adding 0.5 mg/ml MTT (3-[4,5-dimethylthiazol-2,5-diphenyltetrazolium bromide) in α-MEM. Following a 45-min incubation, the MTT medium was removed and cells were dissolved with 100 µl/well of 0.5% SDS, 25 mM HCl in 90% isopropanol. A plate reader was used to read the A_570_ values for each well, and percent viability was calculated relative to wells that were not treated with toxin.

### Toxin-induced ADP-ribosylation of eEF-2

The detection of toxin-induced ADP-ribosylation of eEF-2 in intact cells was performed as previously described [18]. Briefly, cells were treated with toxin and cell lysates were prepared using RIPA lysis buffer (1% Nonidet P40, 0.5% sodium deoxycholate and 0.1% SDS in PBS) containing Complete protease inhibitor cocktail (Roche Diagnostics, Indianapolis, IN). Protein concentrations were estimated using BCA reagent and equal amount of proteins were subjected to native PAGE followed by western blotting. Membranes were probed with anti-eEF-2 antibodies.

Alternatively, to assess ADP-ribosylation of eEF-2 in extracts of cells previously treated with toxin, the eEF-2 that was not already modified was quantitated by reaction with biotinylated NAD [9]. In brief, cells were lysed in RIPA buffer containing protease inhibitors and cell lysate (50 µg) was incubated with 100 ng of DT in ADP-ribosylation buffer (20 mM Tris-HCl, pH 7.4; 1 mM EDTA; 50 mM DTT) with 5 µM 6-Biotin-17-NAD (Trevigen) for 30 min at 25°C. Samples were subjected to SDS-PAGE followed by western blotting with streptavidin-IR conjugate (Rockland Immunochemicals, Gilbertsville, PA) to detect biotin-ADP-ribose-eEF-2.

### Imaging Beta-Lactamase Activity in Cells

Activity of beta-lactamase was measured using the fluorogenic substrate, CCF2/AM (Invitrogen) as described previously [20], [21]. Cells were sub-cultured in 6-well plates one day prior to treatment with toxin. Cells were treated with PA and LFnBLA (2 µg/ml each) for 1 h at 37°C and fresh media replaced the toxin medium. CCF2/AM substrate was prepared as a 6X solution by mixing three components of the kit as mentioned in the manufacturer's instructions. For loading the cells, substrate was diluted directly in the medium for a final concentration of 1.5 µM and incubated for 60 min at room temperature and then replaced with fresh medium. Cells were incubated for an additional 1 h at room temperature to allow FRET disruption. Cells were then visualized and photographed using a Zeiss Axioplan inverted microscope with Zeiss Axiovision software (Carl Zeiss, Jena, Germany). For acquisition of blue fluorescence, excitation filter HQ405/20 nm bandpass, dichroic 425DCXR, and emitter filter HQ460/40 nm bandpass were used. For green fluorescence, HQ405/20 nm bandpass, dichroic 425DCXR, and emitter filter HQ530/30 nm bandpass were used. All filters and dichroic mirrors were purchased from Chroma Technology (Rockingham, VT). All images were acquired using the identical settings.

### Genotyping of eEF-2 Alleles

RNA was extracted using TRIZol reagent (Invitrogen) as per the guidelines. cDNA was synthesized using Superscript (Ambion, now part of Applied Biosystems, Austin, TX) according to the instructions. RT-PCR was carried out to amplify the eEF-2 cDNA. Forward primer 5′-GTCGCCCAACAAGCACAACCG-3′ and reverse primer 5′-GGGACTGT GGATCCCTAATGATGATGATGATGATGCAGTTTGTCCAGGAAGTTGTCCAGT-3′ were used to amplify a 796-bp product of eEF-2 cDNA. An annealing temperature used for PCR was 61°C. The RT-PCR product was purified with PureLink PCR purification kit (Invitrogen, Carlsbad, CA) and digested with MboII restriction enzyme for 2 h. Digested DNA samples were subjected to agarose gel electrophoresis to identify the size of DNA fragments.

## Supporting Information

File S1 Supplementary materials text.
(0.06 MB DOC)

Figure S1 Effect of PA mutants and inhibitors on toxin-induced ADP-ribosylation of eEF-2 in CHO WTP4 and CHO PR328 cells. (A) Cells were treated with FP59 in combination with PA or PA mutants (each 100 ng/ml) for 1 h and then replaced with fresh media. For inhibitor studies, cells were treated with PA + FP59 in the presence of inhibitors (dominant negative mutant of PA, ammonium chloride, or bafilomycin A1) for 1 h and then fresh media replaced toxin. For inhibitors studies with ammonium chloride and bafilomycin, cells were pre-incubated with the respective inhibitors and same inhibitor concentration was maintained during and post-toxin treatment. After a further incubation of 3 h, cell lysates were prepared and equal amounts of protein were loaded on native PAGE followed by western blotting. The membrane was probed with antibody against the carboxy-terminus of eEF-2 of human origin and scanned on Infrared Imager. (B) Toxin-induced ADP-ribosylation in cytosol of CHO PR328 cells. Cells were incubated with FP59 in combination with PA or PA mutant (1 µg/ml each) for 45 min and then fresh media replaced the toxin medium. After an additional incubation of 2.5 h, cytosol was prepared from cells using a hypotonic solution of sucrose. Equal amounts of protein were separated on native PAGE followed by blotting with antibody against the carboxy-terminus of eEF-2 of human origin. Lower panel shows the SDS-PAGE and western blot of same samples and same antibodies to show the equal amount of proteins.
(0.60 MB PPT)

Figure S2 Anthrax lethal toxin-induced cleavage of MEK1 in CHO WTP4 and CHO PR328 cells. Cells were treated with PA + LF (1 µg/ml each) for indicated time periods and then lysates were prepared using RIPA buffer having protease inhibitors. Equal amounts of samples were subjected to SDS-PAGE and western blotting with anti-MEK1-NT antibodies.
(0.23 MB PPT)

Figure S3 Sequence analysis of eEF-2. Protein sequences for eEF-2 from different organisms were obtained from available databases and aligned using clustal W software. Accession no. for eEF-2 sequences are- Cricetulus griseus (GenBank: AAB60497.1); Rattus norvegicus (NCBI Reference Sequence: NP_058941.1); Homo sapiens (GenBank- CAA35829.1); Drosophila melanogaster (Swiss-Prot: P13060.4); Saccharomyces cerevisiae (NCBI ref. no.: NP_014776.1) and Methanococcus vannielii (Swiss-Prot: P09604.2). Arrow ( ) points to the His715 residue that is modified to make the diphthamide residue. “*” below the multiple alignment shows the strictly conserved amino acids between different organisms. “ ↓” denote the reported mutation sites (residues no. Ile714, Gly717 and Gly719 in mammalian eEF-2). As is evident, the region around the diphthamide site (diphthamide loop; residues 709–719 in mammalian eEF-2) is quite conserved among various organisms and most of the mutations reported lie within this loop. Only one other mutation (Ser584 in mammalian eEF-2; not shown here) far from this region is reported, but based on the three-dimensional structure of homologous proteins this residue is also located very close to the diphthamide region.
(0.12 MB PPT)
