# Supplementary material for: Characterization of a Chinese Hamster Ovary Cell Mutant Having a Mutation in Elongation Factor-2
Source: PLoS One. 2010 Feb 5;5(2):e9078. doi: 10.1371/journal.pone.0009078 (PMC2816718; doi:10.1371/journal.pone.0009078)
Supplement: File S1 — Supplementary materials text. (0.06 MB DOC) [file pone.0009078.s001.doc]

**Supplementary materials**

***Additional results and discussion:***

**Effect of PA mutants and inhibitors on ADP-ribosylation of eEF-2**

To examine whether CHO PR328 is altered in toxin uptake and trafficking, we used additional tools to characterize these processes. PA that has bound to cell surface receptors is cleaved by cell surface proteases and the resulting 63-kDa PA fragment (PA63) forms a heptameric pre-pore moiety [1]. After binding of LF or EF, the complex is endocytosed and acidification converts the pre-pore to a functional pore that translocates LF and EF to the cytosol [2]. Many PA mutants have been identified which are defective in some step in this process. The PA delFF mutant in which Phe^313^ and Phe^314^ are deleted cannot form a fully functional membrane channel [3], either because these hydrophobic residues initiate the membrane bilayer insertion event [4] or because they are located at the tip of the inserted beta hairpin [5]. An additional mutant, PA DN, that has been useful in examining the translocation process, is the double mutant PA K397D/D425K. This mutant exerts a dominant negative effect by being incorporated into the heptameric pre-pore and disrupting its translocation activity [6,7]. Cells were treated with PA or PA mutants in combination with FP59 and cell lysates were assessed for ADP-ribosylation of eEF-2. The PA delFF mutant still supported some FP59 internalization and ADP-ribosylation of eEF-2 in both the parental CHO WTP4 and mutant CHO PR328 cells (Fig. S1a), apparently because it was used at a concentration high enough so that its residual activity was sufficient. However, no ADP-ribosylation was observed when PA DN was used (Fig. S1a). Furthermore, as expected, PA DN blocked the function of co-administered wild type PA when the proteins were added at a ratio of 1:1 (Fig. S1a). Both PA mutants produced similar degrees of ADP-ribosylation in CHO WTP4 and CHO PR328 cells, consistent with the data above and supporting the view that the CHO PR328 cells are not altered in toxin trafficking.

Further analyses of toxin trafficking in the mutant CHO cells were done using well-characterized inhibitors of toxin internalization. Several inhibitors have been reported to effectively block internalization of anthrax toxin by preventing acidification of endosomes. Bafilomycin A1 inhibits the vacuolar type H^(+)^ATPase, while ammonium chloride acts as a lysosomotropic agent to elevate the pH of acidic endosomes. Both of these inhibitors are known to block membrane insertion of the PA heptamer and thereby toxin action [8–10]. Use of these inhibitors can also address the question of whether toxin which is endocytosed but not translocated can survive in endosomes and induce ADP-ribosylation of eEF-2 when released during cell lysis. When ADP-ribosylation was assessed by native PAGE and blotting using anti-eEF-2, these inhibitors were found to prevent ADP-ribosylation in both CHO WTP4 and CHO PR328 cells, indicating that internalization of FP59 was similar in both cell types (Fig. S1a). These results also showed that toxin trapped in vesicles cannot induce ADP-ribosylation during cell lysis.

To determine if ADP-ribosylation of eEF-2 can be observed in CHO PR328 cells without disrupting endocytic vesicles, cytosol was prepared after treatment with toxin. An osmotic lysis procedure was used to achieve controlled lysis of the plasma membrane so as to release only cytosol without rupturing internal organelles [11]. A hypotonic solution of sucrose was used for this purpose. Native PAGE was used to analyze the presence of ADP-ribosylated eEF-2 in CHO PR328 cells. ADP-ribosylation of eEF-2 was evident in the cytosol of these cells (Fig. S1b), clearly indicating that ADP-ribosylation is a real *in vivo* event. Moreover, when the PA DN mutant was used in same experiment, no ADP-ribosylation was observed, confirming that translocation of FP59 to the cytosol is required.

**Effect of anthrax lethal toxin in CHO PR328 cells**

CHO cells are resistant to the lytic effects of anthrax lethal toxin but they do exhibit cleavage of MEKs in response to anthrax lethal toxin [12]. Thus, cleavage of MEK1 can be used as an indication of internalization of toxin to the cytosol of CHO cells. To analyze the effect of anthrax lethal toxin, CHO WTP4 and CHO PR328 cells were treated with LF in combination with PA for various time points and cleavage of MEK1 was analyzed by western blotting with anti-MEK1 NT antibodies. These antibodies are against an N-terminal epitope of MEK1 which is cleaved and destroyed by the action of LF [12]. Cleavage of MEK1 was seen to occur with a very similar time course in CHO WTP4 and CHO PR328 cells (Fig. S2), with cleavage being complete by 60 min of toxin treatment.

***Supplementary methods:***

**Effect of PA mutants and inhibitors on toxin induced ADP-ribosylation of eEF2.** In the experiments where inhibitors were used, cells were pretreated with inhibitor and then co-incubated with toxin before preparing cell lysates. Bafilomycin A1 and ammonium chloride were used at final concentrations of 10 nM and 30 mM, respectively. For analyses of cytosolic content, hypotonic lysis of the plasma membrane was achieved by sucrose lysis buffer (250 mM sucrose, 3 mM imidazole, pH 7.4) [11]. Cell pellet was re-suspended in 1.5 ml of lysis buffer containing EDTA-free Complete protease inhibitor cocktail and incubated on ice for 10 min. Samples were centrifuged at 2500 rpm for 10 min at 4°C, and the resultant pellet was re-suspended in 200 µl of lysis buffer followed by passage through a 21-gauge needle. Lysate was then centrifuged at 2500 rpm and supernatant was subjected to another round of centrifugation at 45000 rpm. The resulting supernatant was used as the cytoplasmic fraction and protein content was estimated using BCA protein assay (Thermo Fisher Scientific, Rockford, IL). Samples were then subjected to western blotting and probed with anti-eEF-2 antibodies (1:1000).

**Anthrax lethal toxin induced cleavage of MEK1.** Cells were treated with PA and LF for various time points and washed three times with DPBS before lysates were prepared using RIPA buffer. Protein concentration of lysates was determined using BCA protein assay and equal amounts were loaded on SDS-PAGE. Western blotting was performed using anti-MEK1 NT which detects the N-terminal epitope of MEK1 (this epitope is removed by LF treatment) [12].

References for supporting information

1. Leppla SH (2006) *Bacillus anthracis* toxins. In: Alouf JE, Popoff MR, editors. The Comprehensive Sourcebook of Bacterial Protein Toxins. Burlington, MA: Academic Press. pp. 323-347.

2. Collier RJ, Young JAT (2003) Anthrax toxin. Ann Rev Cell Dev Biol 19: 45-70.

3. Singh Y, Klimpel KR, Arora N, Sharma M, Leppla SH (1994) The chymotrypsin-sensitive site, FFD315, in anthrax toxin protective antigen is required for translocation of lethal factor. J Biol Chem 269: 29039-29046.

4. Wang J, Vernier G, Fischer A, Collier RJ (2009) Functions of phenylalanine residues within the beta-barrel stem of the anthrax toxin pore. PLoS ONE 4: e6280.

5. Petosa C, Collier RJ, Klimpel KR, Leppla SH, Liddington RC (1997) Crystal structure of the anthrax toxin protective antigen. Nature 385: 833-838.

6. Sellman BR, Mourez M, Collier RJ (2001) Dominant-negative mutants of a toxin subunit: an approach to therapy of anthrax. Science 292: 695-697.

7. Yan M, Collier RJ (2003) Characterization of dominant-negative forms of anthrax protective antigen. Mol Med 9: 46-51.

8. Friedlander AM (1986) Macrophages are sensitive to anthrax lethal toxin through an acid-dependent process. J Biol Chem 261: 7123-7126.

9. Gordon VM, Leppla SH, Hewlett EL (1988) Inhibitors of receptor-mediated endocytosis block the entry of *Bacillus anthracis* adenylate cyclase toxin but not that of *Bordetella pertussis* adenylate cyclase toxin. Infect Immun 56: 1066-1069.

10. Dal Molin F, Tonello F, Ladant D, Zornetta I, Zamparo I, et al. (2006) Cell entry and cAMP imaging of anthrax edema toxin. EMBO J 15: 5405-5413.

11. Wickliffe KE, Leppla SH, Moayeri M (2008) Killing of macrophages by anthrax lethal toxin: involvement of the N-end rule pathway. Cell Microbiol 10: 1352-1362.

12. Liu S, Leppla SH (2003) Cell surface tumor endothelium marker 8 cytoplasmic tail-independent anthrax toxin binding, proteolytic processing, oligomer formation, and internalization. J Biol Chem 278: 5227-5234.
