# Supplementary material for: Characterization of a Chinese Hamster Ovary Cell Mutant Having a Mutation in Elongation Factor-2
Source: PLoS One. 2010 Feb 5;5(2):e9078. doi: 10.1371/journal.pone.0009078 (PMC2816718; doi:10.1371/journal.pone.0009078)
Supplement: Figure S1 — Effect of PA mutants and inhibitors on toxin-induced ADP-ribosylation of eEF-2 in CHO WTP4 and CHO PR328 cells. (A) Cells were treated with FP59 in combination with PA or PA mutants (each 100 ng/ml) for 1 h and then replaced with fresh media. For inhibitor studies, cells were treated with PA + FP59 in the presence of inhibitors (dominant negative mutant of PA, ammonium chloride, or bafilomycin A1) for 1 h and then fresh media replaced toxin. For inhibitors studies with ammonium chloride and bafilomycin, cells were pre-incubated with the respective inhibitors and same inhibitor concentration was maintained during and post-toxin treatment. After a further incubation of 3 h, cell lysates were prepared and equal amounts of protein were loaded on native PAGE followed by western blotting. The membrane was probed with antibody against the carboxy-terminus of eEF-2 of human origin and scanned on Infrared Imager. (B) Toxin-induced ADP-ribosylation in cytosol of CHO PR328 cells. Cells were incubated with FP59 in combination with PA or PA mutant (1 µg/ml each) for 45 min and then fresh media replaced the toxin medium. After an additional incubation of 2.5 h, cytosol was prepared from cells using a hypotonic solution of sucrose. Equal amounts of protein were separated on native PAGE followed by blotting with antibody against the carboxy-terminus of eEF-2 of human origin. Lower panel shows the SDS-PAGE and western blot of same samples and same antibodies to show the equal amount of proteins. (0.60 MB PPT) [file pone.0009078.s002.ppt]

## Slide 1
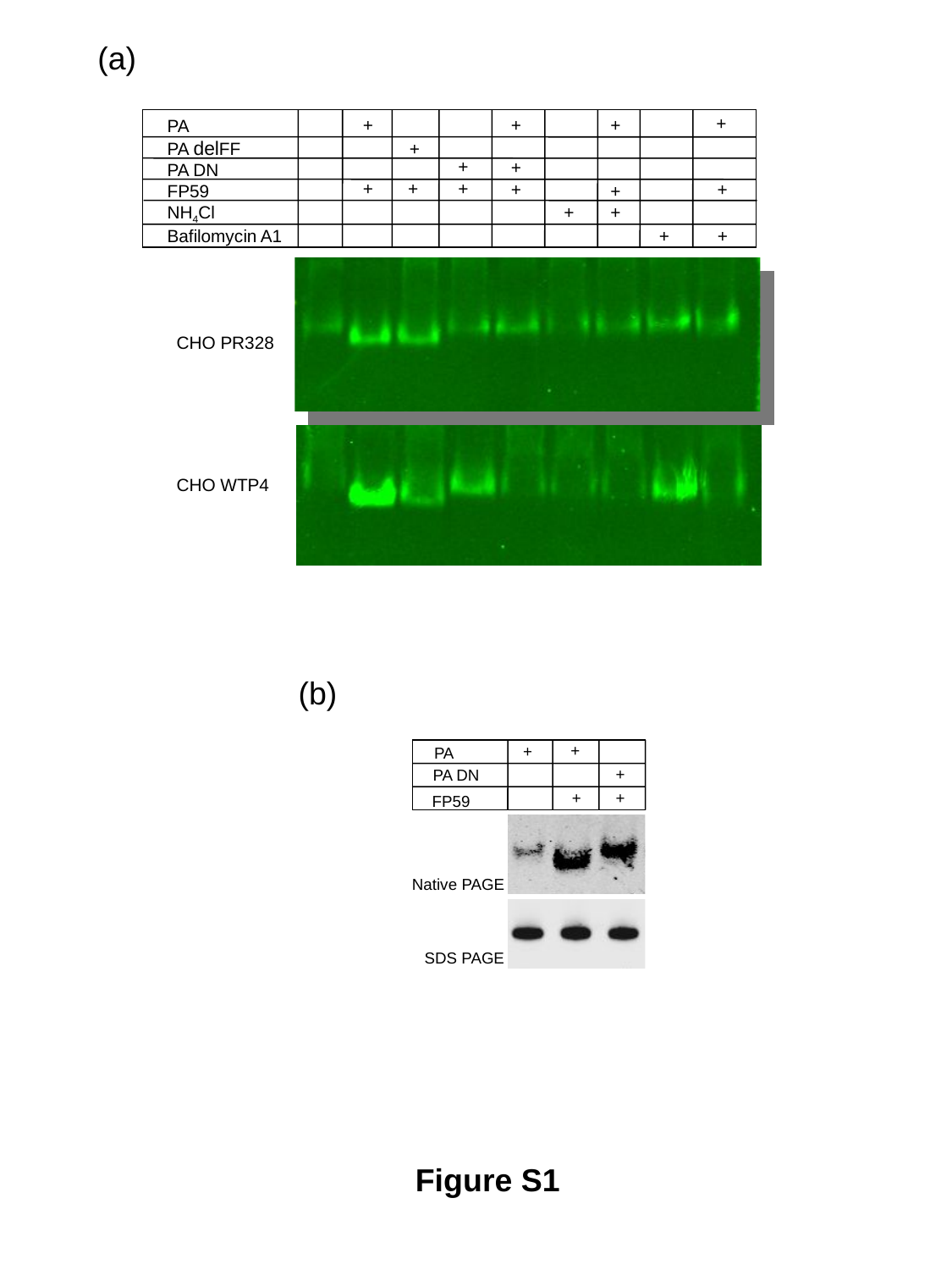

(a)
+
+
+
+
PA
PA delFF
PA DN
FP59
NH4Cl
Bafilomycin A1
+
+
+
+
+
+
+
+
+
+
+
+
+
CHO PR328
CHO WTP4
(b)
+
+
 PA
+
 PA DN
+
+
 FP59
Native PAGE
SDS PAGE
Figure S1
