# Supplementary figures and images for: Characterization of a Chinese Hamster Ovary Cell Mutant Having a Mutation in Elongation Factor-2
Source: PLoS One. 2010 Feb 5;5(2):e9078. doi: 10.1371/journal.pone.0009078 (PMC2816718; doi:10.1371/journal.pone.0009078)

## Slide 1
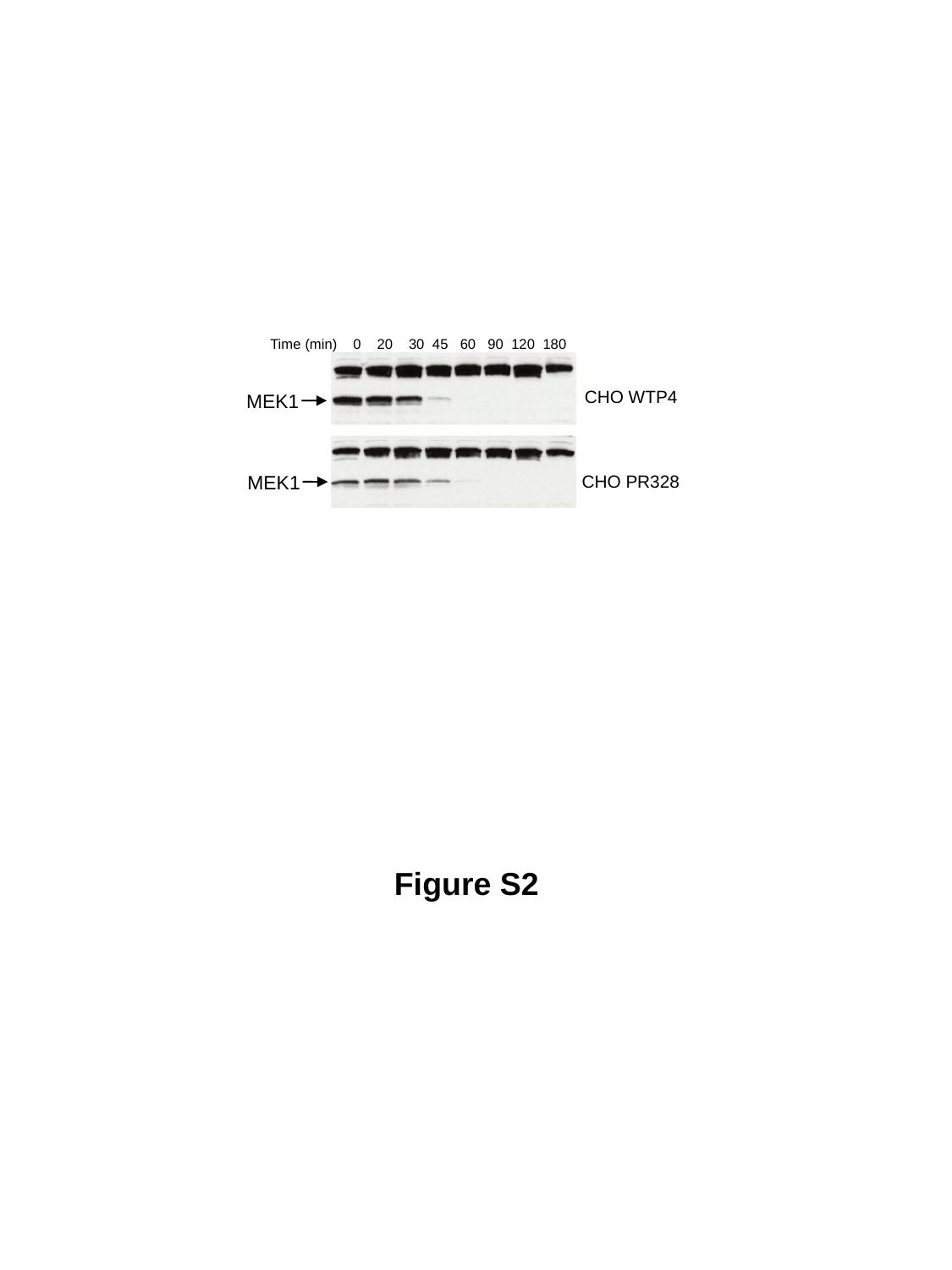

Time (min) 0 20 30 45 60 90 120 180
CHO WTP4
MEK1
MEK1
CHO PR328
Figure S2

Supplement: Figure S2 — Anthrax lethal toxin-induced cleavage of MEK1 in CHO WTP4 and CHO PR328 cells. Cells were treated with PA + LF (1 µg/ml each) for indicated time periods and then lysates were prepared using RIPA buffer having protease inhibitors. Equal amounts of samples were subjected to SDS-PAGE and western blotting with anti-MEK1-NT antibodies. (0.23 MB PPT) [file pone.0009078.s003.ppt]

## Slide 1
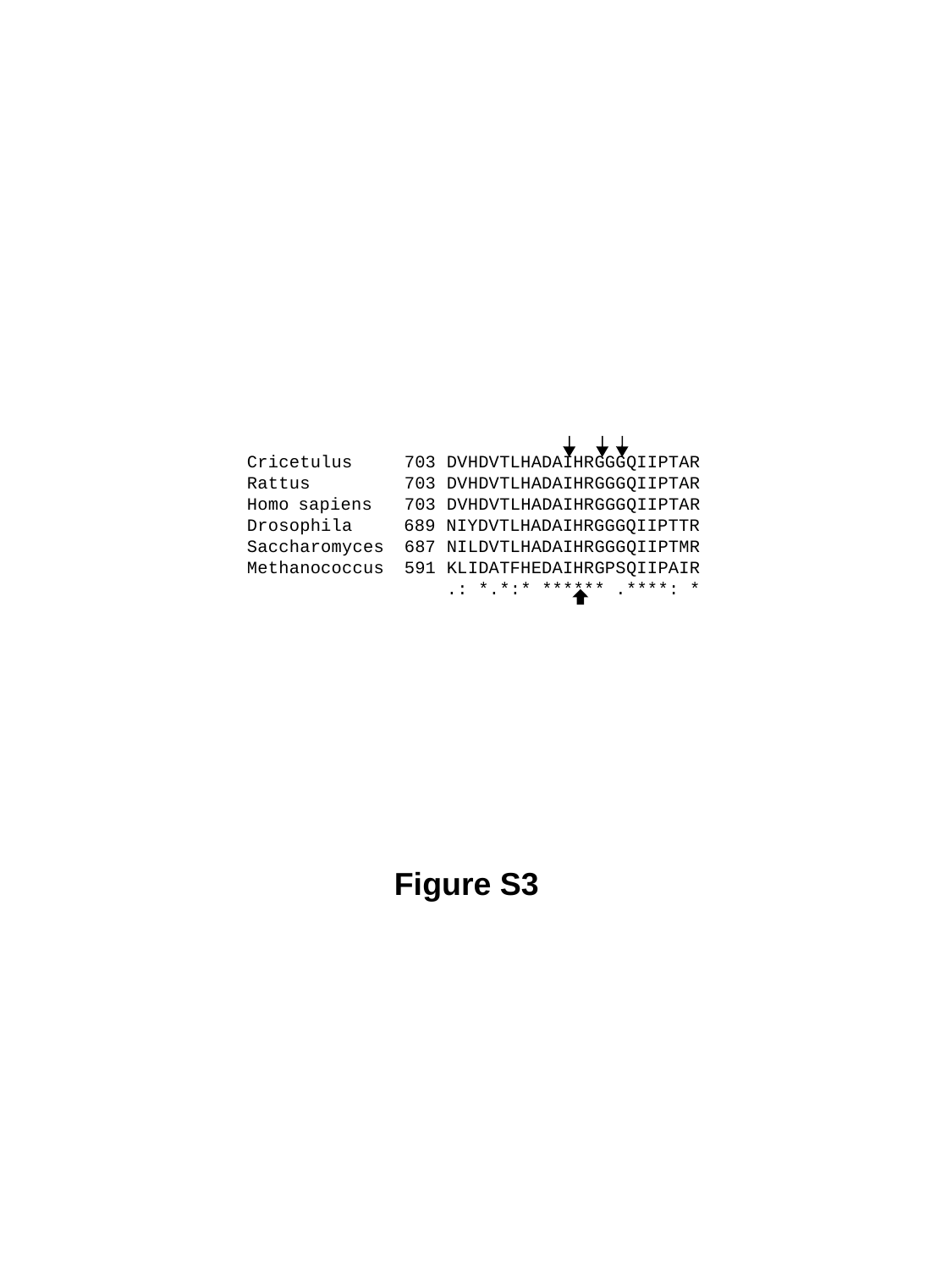

Figure S3

Supplement: Figure S3 — Sequence analysis of eEF-2. Protein sequences for eEF-2 from different organisms were obtained from available databases and aligned using clustal W software. Accession no. for eEF-2 sequences are- Cricetulus griseus (GenBank: AAB60497.1); Rattus norvegicus (NCBI Reference Sequence: NP_058941.1); Homo sapiens (GenBank- CAA35829.1); Drosophila melanogaster (Swiss-Prot: P13060.4); Saccharomyces cerevisiae (NCBI ref. no.: NP_014776.1) and Methanococcus vannielii (Swiss-Prot: P09604.2). Arrow ( ) points to the His715 residue that is modified to make the diphthamide residue. “*” below the multiple alignment shows the strictly conserved amino acids between different organisms. “ ↓” denote the reported mutation sites (residues no. Ile714, Gly717 and Gly719 in mammalian eEF-2). As is evident, the region around the diphthamide site (diphthamide loop; residues 709–719 in mammalian eEF-2) is quite conserved among various organisms and most of the mutations reported lie within this loop. Only one other mutation (Ser584 in mammalian eEF-2; not shown here) far from this region is reported, but based on the three-dimensional structure of homologous proteins this residue is also located very close to the diphthamide region. (0.12 MB PPT) [file pone.0009078.s004.ppt]
